# Cardiac Involvement In Multiorgan Sarcoidosis: Prognostic and Therapeutic Implications

**DOI:** 10.7759/cureus.10714

**Published:** 2020-09-29

**Authors:** Cecilia Higueruela-Mínguez, Ana Martín-García, Antonio J Chamorro, Miguel Marcos, Silvio Ragozzino

**Affiliations:** 1 Internal Medicine, University Hospital of Salamanca, Salamanca, ESP; 2 Cardiology, University Hospital and University of Salamanca, Salamanca, ESP; 3 Cardiology, Institute of Biomedical Research of Salamanca (IBSAL), Salamanca, ESP; 4 Centro de Investigación Biomédica en Red Enfermedades Cardiovasculares (CIBERCV), Instituto de Salud Carlos III, Madrid, ESP; 5 Internal Medicine, Institute of Biomedical Research of Salamanca (IBSAL), Salamanca, ESP

**Keywords:** cardiac sarcoidosis, multiorgan sarcoidosis, cardiac magnetic resonance, prognosis, steroid therapy

## Abstract

Sarcoidosis is a systemic granulomatous disease with a highly variable clinical impact. Accurate prognostic evaluation is fundamental to establish the best therapeutic approach. Multiorgan disease and especially the involvement of vital organs, such as the heart, are associated with worse outcomes and often require more aggressive therapy. Here, we describe the case of a young adult with sarcoidosis with lymph node, pulmonary, hepatosplenic, and cardiac involvement. This clinical scenario emphasizes the importance of a thorough prognostic assessment and highlights some of the main unmet clinical needs for the risk stratification and management of these patients.

## Introduction

Sarcoidosis is a systemic granulomatous disease of unknown etiology that typically affects young adults. It was first described in 1877, but medical and anthropological research has recently led some authors to propose a retrospective diagnosis of multiorgan sarcoidosis for Maximilien de Robespierre (1758-1794), one of the most influential figures of the French Revolution. Therefore, this could represent the oldest known case of sarcoidosis [1].

Sarcoidosis has a variable clinical impact, ranging from an asymptomatic state with a self-limiting course to a life-threatening condition. The disease most frequently presents with pulmonary and/or lymph node involvement, but any organ may be affected [2]. Multiorgan sarcoidosis, defined as the involvement of three or more organs, is present in approximately 20% of patients with sarcoidosis and is generally associated with a poorer prognosis [3]. Nevertheless, the assessment of the clinical burden in terms of morbidity and mortality and the subsequent determination of the need for immunosuppressive therapy depend on the degree of functional impairment and the involvement of vital organs [4]. For example, eye involvement implies no mortality risk but can lead to vision loss. On the other hand, cardiac sarcoidosis can be clinically silent in most patients, but initial manifestations, such as ventricular arrhythmia, heart block, and sudden death, may be lethal [5]. These observations emphasize the need for an accurate diagnostic assessment and thorough risk stratification of patients with sarcoidosis to establish the best therapeutic approach. Cardiac involvement has a low reported incidence (detected in about 5% of all patients with sarcoidosis) [6], but postmortem studies have revealed that its true prevalence is more than 25% [7]. Advanced diagnostic tools, with high sensitivity in the detection of cardiac sarcoidosis, include cardiac magnetic resonance imaging (CMRI) and positron emission tomography (PET) [8]. Whether all patients with sarcoidosis should be screened for cardiac involvement, and whether the treatment of asymptomatic individuals with such involvement, is beneficial remains questionable. Here, we present a clinical scenario that highlights some of the knowledge gaps that currently affect prognostic evaluation and therapeutic selection for patients with multiorgan sarcoidosis.

## Case presentation

A 31-year-old man presented to the emergency department of our hospital complaining of abdominal pain. The pain was located in the right upper quadrant, constant, and non-radiating. The patient also reported mild dyspnea on exertion in previous months. He reported no other symptom. The patient’s medical history included an episode of acute pericarditis five years previously. He was a mild smoker (2-3 cigarettes per day) and worked as a farmer. On examination, bibasilar dry crackles and tenderness in the right upper quadrant were detected. Blood analysis showed no relevant abnormality, except for slight increases in the gamma-glutamyl transferase and C-reactive protein concentrations (Table 1).

**Table 1:** Main laboratory findings, at presentation and after treatment ACE: Angiotensin-Converting Enzyme; ALT: Alanine Aminotransferase; AST: Aspartate Aminotransferase; CK-MB: Creatine Kinase; CRP: C-Reactive Protein; cTnT: Cardiac Troponin T; GGT: Gamma-Glutamyl Transpeptidase; ESR: Erythrocyte Sedimentation Rate; NA: Not Available

Blood tests, unit (reference range)	At presentation	After treatment
Leucocytes, x 10^3^/mL (4.5 – 11.0)	5.2	5.1
Neutrophils (1.8 - 7.7)	3.09	3.37
Lymphocytes (1.0 - 4.8)	0.88	0.75
AST, U/L (0 - 40)	21	15
ALT, U/L (0 - 41)	28	30
GGT, U/L (0 - 60)	121	62
CRP, mg/dL (0 - 0.5)	2.98	0.32
ESR, mm/h (0 - 25)	17	14
ACE, U/L (27 - 68)	197	44
Calcemia, mg/dL (8.9 - 10.4)	9.9	9.3
24h Calciuria, mg/24h (100 - 300)	577.6	NA
CK, U/L (25 - 190)	53	64
cTnT, pg/mL (0 - 14)	3	NA

**Figure 1:**
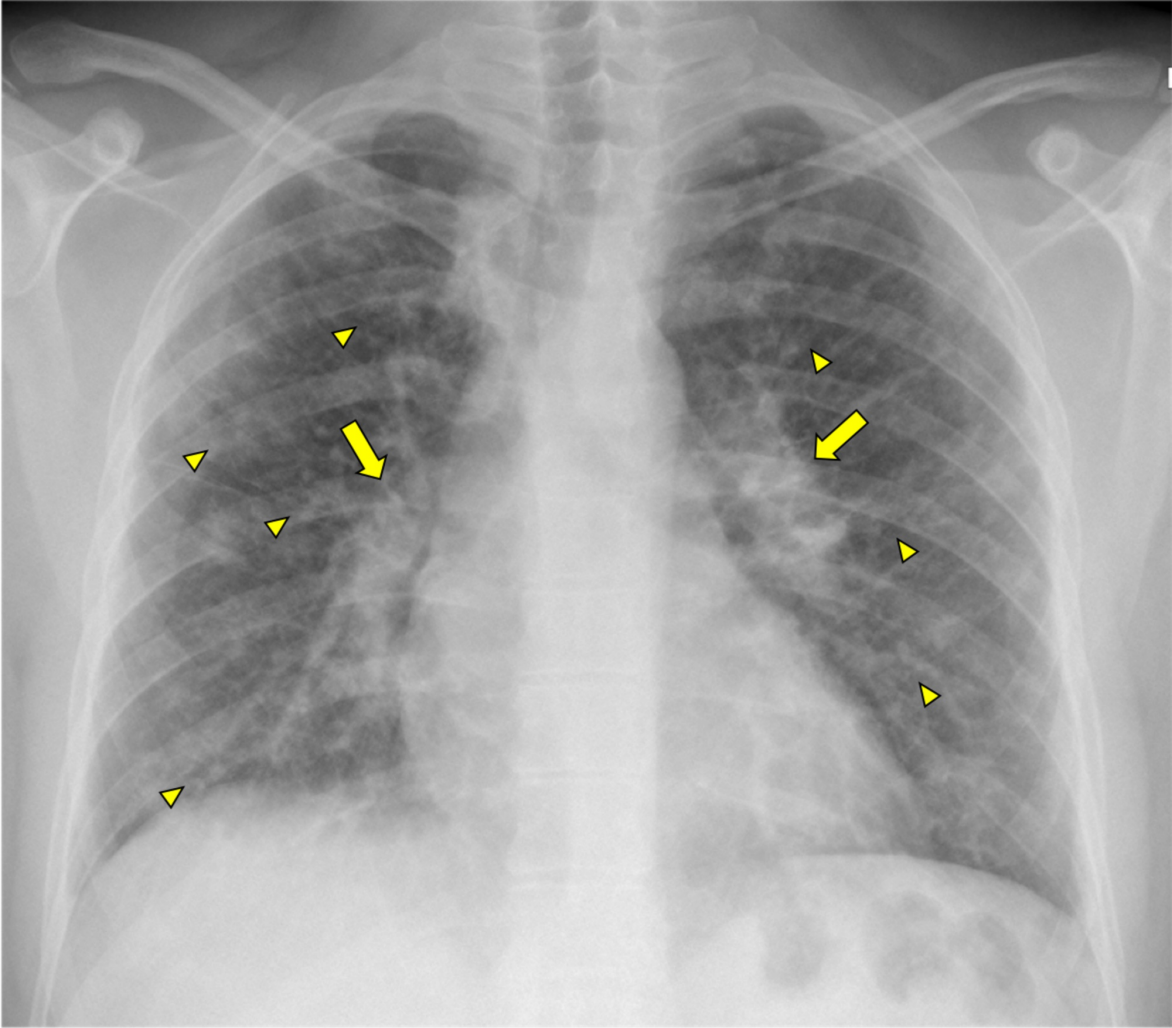
Chest X-ray: bilateral hilar enlargement (arrows) and reticulonodular opacities (arrowheads)

**Figure 2:**
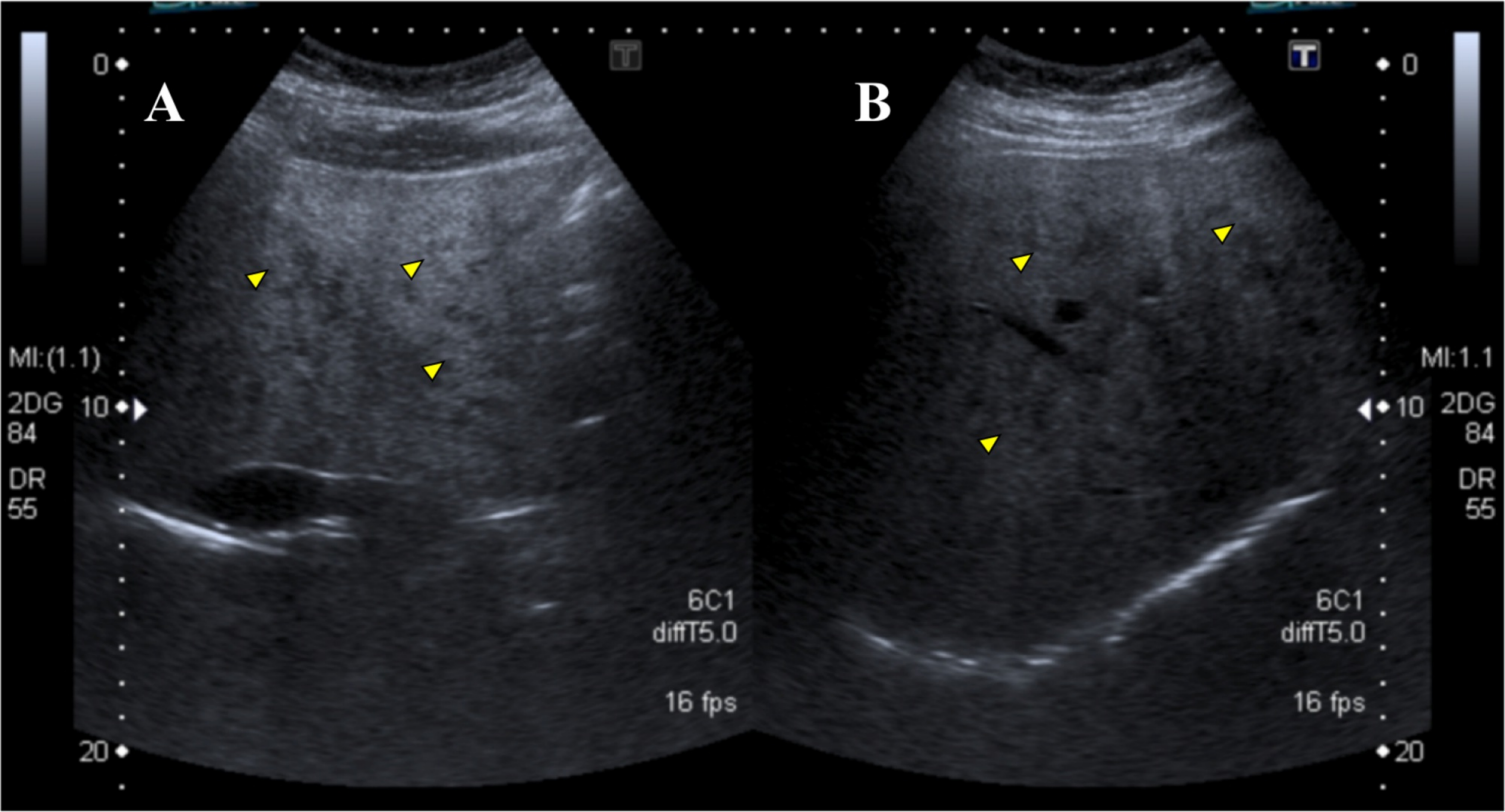
Abdominal ultrasound: diffuse hepatic micronodular involvement (arrowheads) (A) Epigastric transverse scanning. (B) Right subcostal scanning

Chest X-ray demonstrated bilateral hilar enlargement and reticulonodular opacities (Figure 1), and abdominal ultrasound showed diffuse micronodular hepatic involvement (Figures 2A-2B).

**Figure 3:**
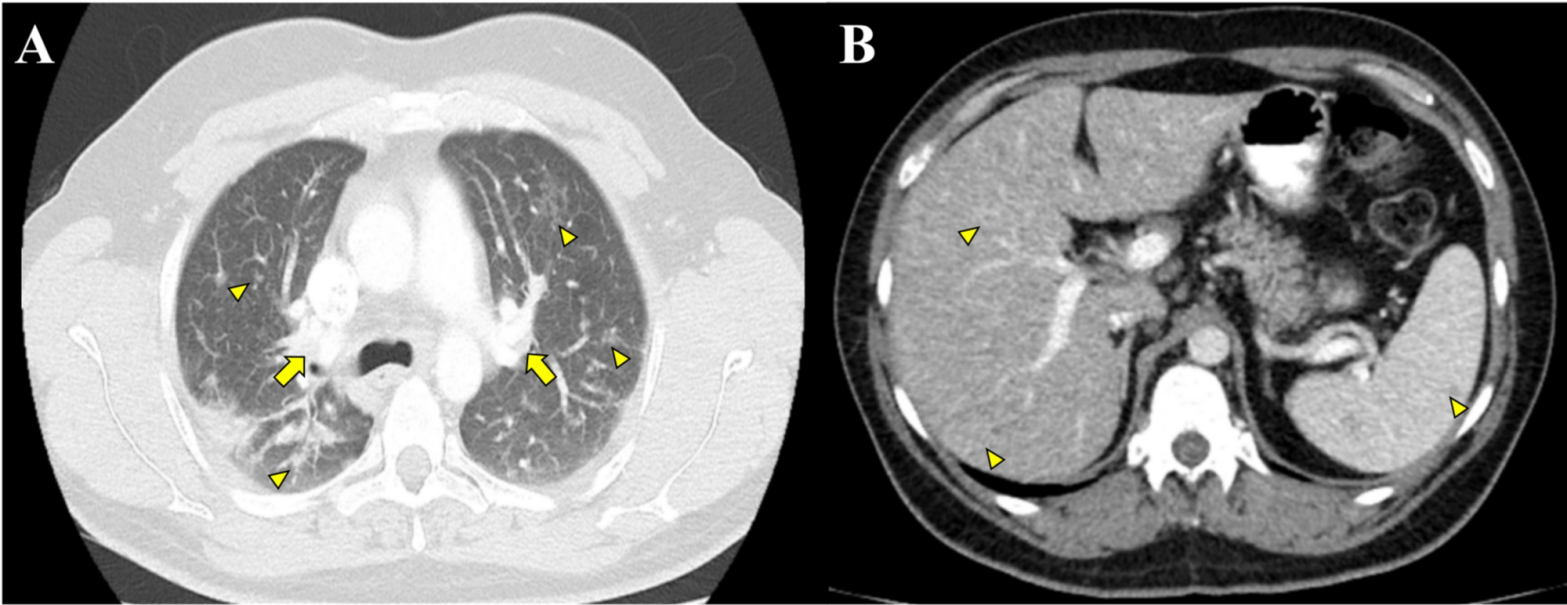
Whole-body computed tomography. (A) Enlargement of hilar lymph nodes (arrows) and micronodular pattern (arrowheads). (B) Diffuse hepatic and splenic involvement (arrowheads).

As the above-mentioned findings were suggestive of granulomatous disease, a diagnostic workup was started: the differential diagnosis at this stage was broad, including infectious diseases, especially mycobacterial and fungal infections; neoplastic diseases, such as lymphoma, systemic diseases, hypersensitivity, and foreign body granulomatosis. Further laboratory and imaging tests were performed: an elevated angiotensin-converting enzyme (ACE) level and hypercalciuria led to the suspicion of sarcoidosis (Table 1). Whole-body computed tomography revealed splenic involvement in addition to the prior findings (Figures 3A-3B).

**Figure 4:**
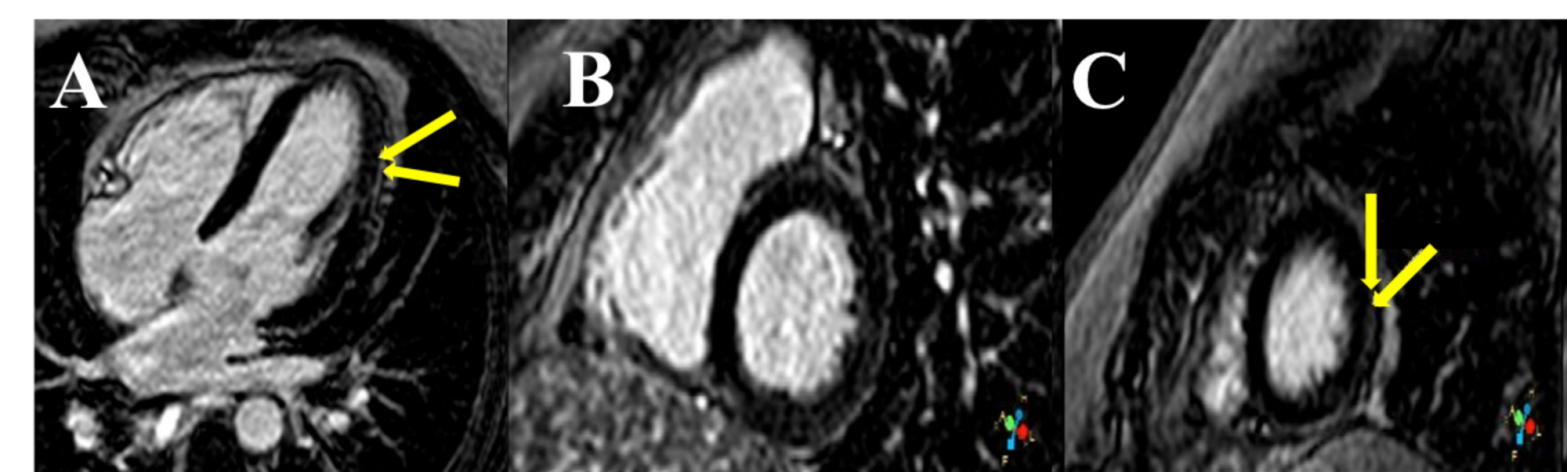
Late gadolinium enhancement cardiac magnetic resonance images: subepicardial delayed enhancement at the lateral left ventricular wall (arrows) (A) Long-axis view. (B) Short-axis view, basal segment. (C) Short-axis view, apical segment

Human immunodeficiency virus and tuberculin skin tests, microbiological cultures, and autoimmunity laboratory tests were negative. The results of bronchoscopy with bronchoalveolar lavage and bronchial biopsy were inconclusive. Thus, a liver biopsy showing non-caseating granulomas was needed to confirm the diagnosis. Lung function tests showed a mild restrictive pattern with a slight decrease in the diffusing capacity for carbon monoxide. To rule out other extrapulmonary involvement, further tests, such as electrocardiography (ECG), 24-h Holter ECG monitoring, and ocular examinations, including tonometry and slit-lamp and fundoscopic testing, were performed. Findings of a basic cardiac evaluation were normal, but those of CMRI were consistent with the diagnosis of cardiac sarcoidosis: T2-weighted sequences showed apical edema and subepicardial delayed enhancement at the lateral left ventricular wall (Figures 4A-4C).

The final diagnosis was sarcoidosis with lymph node, pulmonary, hepatosplenic, and cardiac involvement. The patient was started on prednisone (1 mg/kg/day). A short-term follow-up visit was scheduled to monitor the therapeutic response and adverse effects of the corticosteroid and to plan the gradual tapering of the steroid dosage. At six months, the patient’s ACE level had normalized (Table 1) and imaging tests showed a clear improvement of the lung and cardiac involvement. After two years of follow-up, the patient is on prednisone (5 mg/day) and is feeling well.

## Discussion

We presented a case of multiorgan sarcoidosis with lymph-node, pulmonary, hepatosplenic, and cardiac involvement. Sarcoidosis is often a challenging diagnosis for clinicians. Diagnostic criteria include clinical and radiological presentations, evidence of non-caseating granulomas, and the exclusion of alternative diseases [2]. Biopsy sampling is not necessary with specific presentations such as Löfgren’s syndrome, Heerfordt’s syndrome, and typical bilateral hilar lymphadenopathy. Otherwise, histological proof of the presence of non-caseating epithelioid granulomas significantly improves the reliability of the diagnosis [2]. Although radiological and laboratory test findings were suggestive of the diagnosis of sarcoidosis in our patient, the clinical relevance and therapeutic implications of some alternative diagnoses led us to obtain histological confirmation by liver biopsy.

Sarcoidosis may affect almost every organ, and the prognosis depends on the extent and severity of the disease, as well as the involvement of vital organs, such as the brain and heart [4]. Indeed, the presence of multisystemic manifestations with extrathoracic dissemination is associated with worse outcomes and more often with the need for chronic treatment [9]. Thus, when sarcoidosis is suspected or confirmed, a comprehensive evaluation of symptoms and signs that can suggest extrapulmonary involvement is important. The case described here was characterized by a dissociation between the clinical presentation and the radiological extent of the disease. The patient was paucisymptomatic, with the main complaint of abdominal pain, which was difficult to relate to sarcoidosis. He only reported mild dyspnea on exertion when we inquired about it specifically. Multiorgan sarcoidosis was detected almost incidentally on initial assessment, with lymph-node, pulmonary, and hepatosplenic involvement. Although no significant functional impairment was identified, several questions arose about the appropriate management of this case. Is such extensive involvement a valid reason for starting immunosuppressive therapy or should clinicians “wait and see” in such cases? Is the risk of involvement of other organs greater in such a patient? Should clinicians actively look for the involvement of other organs, regardless of the existence of symptoms?

These questions mainly arise because multiorgan sarcoidosis is considered to be a severe clinical phenotype of sarcoidosis, with a greater risk of poorer outcomes [4]. Nevertheless, due to the lack of evidence, no specific recommendation can be established for this group of patients. Decisions about whether to start treatment are generally guided by three main criteria: the risk of dysfunction or irreversible damage to a major organ, the presence of incapacitating symptoms, and the mortality risk [2]. Based on these factors, no element of our clinical scenario firmly indicated the initiation of immunosuppressive therapy. For this reason, and to ensure thorough prognostic evaluation, we decided to rule out the presence of cardiac involvement.

Cardiac sarcoidosis is classically thought to be uncommon, and it is diagnosed only in 5% of living patients with sarcoidosis [6]. However, autopsy series have demonstrated that more than one in four patients with sarcoidosis had myocardial granulomas [7]. Cardiac involvement is a poor prognostic factor. For instance, a Japanese series suggested that it is responsible for up to 85% of the deaths of patients with sarcoidosis [10]. Clinical manifestations vary widely: myocardial infiltration and fibrosis may provoke conduction abnormalities, such as atrioventricular block, atrial and ventricular arrhythmias, heart failure, and sudden cardiac death. Nevertheless, up to 55% of patients are completely asymptomatic [11]. Thus, a high index of suspicion of this clinical entity is required to reach the diagnosis. Patients with sarcoidosis should always undergo comprehensive cardiac history taking and physical examinations, as well as 12-lead ECG and echocardiography [2]. Our patient had no cardiovascular symptoms, except for mild dyspnea and an episode of pericarditis five years previously. Findings of the initial basic cardiac assessment were negative; importantly, ECG abnormalities are present in less than 10% of cases of clinically silent cardiac sarcoidosis [12], and echocardiographic findings manifest only in a late clinical stage [13]. Endomyocardial biopsy has high specificity but lacks sensitivity (probably due to patchy involvement), and it is an invasive procedure [14]. Among non-invasive tools for the evaluation of cardiac sarcoidosis, CMRI has a very high negative predictive value and sensitivity (virtually 100%), and a specificity of 78%, with a lower false-positive rate than does PET [15]. Further, CMRI findings have prognostic value, as the presence and extent of late gadolinium enhancement are good predictors of cardiac-related death and cardiac-related adverse events [16]. Moreover, CMRI may be useful in assessing the efficacy of therapy [11]. In our case, the detection of cardiac involvement by CMRI definitively tipped the balance in favor of the institution of a more aggressive approach.

Corticosteroids are considered to be the cornerstone of sarcoidosis treatment. However, most data on the therapeutic management of cardiac sarcoidosis are from small observational studies, and no clear guideline on the timing, intensity, or duration of therapy has been established. Despite the paucity of evidence, some experts have suggested an initial dose of 1 mg/kg/day, but lower doses (30-40 mg/day) have been shown to be equally effective in observational studies [2,17]. A minimum of six-12 months of maintenance therapy is generally advised, and the monitoring of disease activity by cardiac imaging should drive decisions on treatment adjustment [2]. The use of corticoid-sparing agents, alone or in combination with steroids, remains controversial. A recent retrospective study showed that the combined use of corticosteroids and immunosuppressive drugs is more effective than the use of steroids alone in preventing a relapse of cardiac sarcoidosis [18].

## Conclusions

The clinical scenario described herein highlights the relevance of cardiac screening for patients with sarcoidosis and extrathoracic involvement, as well as the diagnostic accuracy of CMRI in this setting. Due to the lack of evidence, further studies are needed to properly establish a risk stratification method for patients with multiorgan sarcoidosis, to enable the identification of patients who will benefit most from CMRI screening and early institution of therapy and to define the best therapeutic approach.
